# Dry eye, its clinical subtypes and associated factors in healthy pregnancy: A cross-sectional study

**DOI:** 10.1371/journal.pone.0258233

**Published:** 2021-10-07

**Authors:** Kofi Asiedu, Samuel Kyei, Madison Adanusa, Richard Kobina Dadzie Ephraim, Stephen Animful, Stephen Karim Ali-Baya, Belinda Akorsah, Mabel Antwiwaa Sekyere

**Affiliations:** 1 Eye Clinic, Cosmopolitan Medical Center, North-Dzorwulu, Accra, Ghana; 2 Department of Optometry and Vision Science, School of Allied Health Sciences, College of Health and Allied Sciences, University of Cape Coast, Cape Coast, Ghana; 3 Division of Family Medicine, Directorate of University Health Services, University of Cape Coast, Cape Coast, Ghana; 4 Department of Medical Laboratory, School of Allied Health Sciences, College of Health and Allied Science, University of Cape Coast, Cape Coast, Ghana; Xiamen University, CHINA

## Abstract

The study determined the frequency of dry eye, its clinical subtypes and risk factors among pregnant women. This study was a hospital-based cross-sectional study of pregnant women visiting the antenatal clinic of the University of Cape Coast hospital. Clinical dry eye tests were performed along with the administration of a symptom questionnaire. Frequencies, chi-square analysis and logistic regression analyses were conducted to determine the frequency of dry eye disease, its clinical subtypes and associated factors. The prevalence of dry eye disease among the cohort of pregnant women was 82/201 (40.8% 95% confidence interval 34.3%-47.3%). Among the 82 pregnant women with dry eye disease, the frequencies of the clinical subtypes of dry eye were: evaporative dry eye [15/82(18.3%; 95% CI, 12.2%–25.2%)], aqueous deficient dry eye [10/82(12.2.%; 95% CI, 7.3%–18.3)], mixed dry eye [6/82(7.3%; 95% CI, 3.7%–11.0%)], and unclassified dry eye [51/82(62.2%; 95% CI, 52.4%–72.0%)]. Binary logistic regression analysis showed that the following factors were not significantly associated with dry eye: age, BMI, lipid profile, prolactin level, testosterone level, ocular protection index and blink rate. Only gestational age was significantly associated with dry eye disease in pregnancy. In conclusion, the current study showed that dry eye disease occurs frequently in pregnant women ranging from the first to the third trimester and it is associated with increasing gestational age. The evaporative dry eye was more common compared to the aqueous deficient dry eye, but most dry eye could not be classified.

## Introduction

Dry eye disease is a prevalent ophthalmic disease and it is the most common reason for patients visiting ophthalmic practitioners [1,2]. It is a disease characterized by symptoms of ocular discomfort, ocular surface damage, tear film instability, loss of homeostasis and neurosensory abnormalities [1]. The etiology of dry eye disease is multifactorial and often multiple instigators contribute to the clinical manifestation of the disease [1]. The impact of moderate dry eye on the quality of life is comparable to moderate angina and severe dry eye is as debilitating as dialysis [3,4]. Dry eye disease is frequently associated with psychosomatic symptoms further entrenching its role as a perpetrator of poor quality of life [3].

Pregnancy is not a pathological condition [5], it involves anatomical, physiological, and biochemical changes in the woman’s body [5]. It is known that the quality of life decreases significantly overtime during pregnancy and it is even worse in the case of pathological pregnancy [6]. Pregnancy is associated with depression and other psychosomatic conditions [7]. This implies that pregnancy places women in a vulnerable state of health. Every little effort to alleviate the challenges in pregnancy is critical. Dry eye disease needs not compound the precarious situation pregnant women face daily. This implies that treating and researching dry eye disease in pregnant women is worthwhile. Pregnancy is associated with hormonal and metabolic changes, which alter the physiology of many body tissues [8,9].

During pregnancy, the changes in hormones, metabolism, hemodynamic, vascular and immunological response can affect the eye, especially the ocular surface tissues instigating changes, which may be transient but in some cases permanent [10–12]. Earlier studies have looked at dry eye disease in pregnant women and reported increased symptomatology and decreased tear secretion in pregnant women [13,14]. Nkiru et al. reported that dry eye symptoms and signs peak from the second to the third trimester and subsides in the postpartum period. However, the study excluded the first-trimester pregnant women hence failing to give a clearer picture of the issue [13]. It has also been shown that pregnant women have worse clinical signs of dry eye compared with age-matched non-pregnant women [14].

Notwithstanding, these earlier studies had relatively small sample sizes, which was an obvious limitation [13,14]. Even though the prevalence of dry eye is reported to be high in pregnant women [13], no study has reported on the clinical subtypes of dry eye in pregnant women. The current paradigm shift in dry eye diagnosis from a sequalae-based diagnosis to an etiology-based diagnosis has made the etiology of dry eye the central basis for selecting treatment [15]. The DEWS II report affirms this new approach and implores ophthalmic practitioners to find the etiology of dry eye whether aqueous deficiency, evaporative dry eye, or mixed and unclassifiable forms [1]. Studies have not yet explored the risk factors for dry eye disease in pregnant women. It is unknown whether there are modifiable risk factors that can be modified to mitigate the high occurrence of dry eye disease in pregnancy.

Pregnant women may be unsuitable for traditional dry eye therapies such as topical immunomodulators and corticosteroids; hence, knowledge about the subtypes of dry eye will be important in therapeutic management. Usually, topical ophthalmic medications do not undergo first-pass metabolism in the liver [16], hence there is a potential for an adverse effect on fetuses despite topical administration.

Finding the etiology of dry eye in pregnant women can prevent many therapeutic misadventures and promote targeted therapeutic regimens. Knowledge of modifiable risk factors is in no doubt useful in the prevention and treatment of dry eye disease in pregnancy. This study sought to examine the frequency, risk factors and clinical subtypes of dry eye disease among a cohort of pregnant women.

## Materials and methods

This study was a hospital-based cross-sectional study. Pregnant women attending antenatal care at the University of Cape Coast Hospital were recruited into the study after an invitation. All pregnant women who met the inclusion criteria and were willing to provide written informed consent were recruited to the study. Pregnant women were examined if they did not have any of the following conditions: a history of ocular surgeries, lid margin abnormalities, history of contact lens wear, ocular surface abnormalities, systemic disease (diabetes, Sjogren syndrome, preeclampsia, etc.), and usage of local or systemic medications that can affect tear function. Pregnant women were included in the study if they fulfilled these inclusion criteria and afterward written informed consent was obtained. Only pregnant women with no remarkable systemic disease or psychological condition were recruited into the study. The recruitment into the study followed the tenets of the Declaration of Helsinki for the use of human subjects in the study. This study received the University of Cape Coast institutional review board approval with ethical clearance number UCCIRB/CHAS/2017/49 and official permission was also obtained from the University of Cape Coast Health Directorate before data collection started at the antenatal clinic. A midwife and obstetrician-gynecologist were present, who attended to pregnant women as routinely done and were available to redress any eventualities outside the research team’s scope during the data collection. In all cases, the study’s pros and cons were clearly explained to all participants before obtaining written consent.

### Sample size calculation

G-Power 3.1.9.2 software (Universität Kiel, Germany) was used to calculate the sample size. A total of 178 pregnant women was adequate to detect an effect size of 0.25 with 85% power at an alpha level of 0.05. The minimum sample size was increased by 13% to make up for any consent withdrawal during the study and enhance our statistical power. A total of 201 pregnant women met the inclusion criteria and were finally included in the analysis.

### Clinical assessments

The same clinician performed all clinical measurements. Study subjects completed the ocular surface disease index (OSDI) before the clinical examination of the ocular surface. The examining clinician was not aware of the symptom score. The clinical examination comprised corneal fluorescein staining, blink rate, fluorescein tear breakup time, meibomian gland expressibility, and lid margin assessments with the slit lamp biomicroscope. All clinical assessments were made for each eye. The ocular protection index was computed for each patient using the tear breakup time and blink interval [17]. Lipid profile was performed on the day of eye examination along with information on gravidity which was also collated.

### Tear breakup time

Fluorescein impregnated paper strip (Optitech eye care, Allahabad, India) was moisturized using normal saline. The paper strip was gently applied to the temporal conjunctiva after being completely wetted. The cornea was examined with a slit lamp using cobalt blue light with a yellow barrier filter. The patient was asked to blink twice and then look straight ahead without blinking. The time from the last blink to the first appearance of a dry spot or dark spot was measured three times with the help of a stopwatch. The mean of three measurements was used as tear breakup time [18].

After the fluorescein tear breakup time was measured, the staining of the cornea was graded and recorded. The dry paper strip impregnated with lissamine green (Optitech eye care, Allahabad, India) was completely wetted with normal saline and gently applied to the temporal conjunctiva after the corneal staining was graded with fluorescein. One to two minutes’ time interval was permitted after instilling the lissamine green dye and grading of the conjunctiva was commenced. The Oxford grading scale (0–15) was used for grading the ocular surface staining [19].

### Blink rate

The number of blinks per minute was measured using a stopwatch while participants were reading the visual acuity chart. The assessment was done under a binocular viewing environment at room temperature (24°C) [18,20].

### Meibomian gland expressibility and quality

Tolerable digital pressure was exerted on the lower eyelid’s tarsus to express the centrally placed eight meibomian glands. The number of meibomian glands oozing meibum and the meibum’s clarity was assessed using the slit-lamp biomicroscope. Meibomian gland expressibility (MGE) was graded using the following scoring scheme: 0 = all glands expressing lipids (normal), 1 = three to four producing lipids, 2 = one to two expressing lipids, 3 = no glands yielding lipids [18].

The quality of meibomian gland secretions (QMG) was graded based on the following scoring scheme as follows: 0 = clear lipids easily expressed (normal), 1 = cloudy lipids expressed with minimal pressure, 2 = cloudy lipids with particles and 3 = inspissated lipids (gel-like). The highest score for any expressed gland was taken as the score [18].

### Posterior lid margin findings

Lid margin thickness: 0 = no lid margin thickening, 1 = lid margin thickening with or without focal rounding, and 2 = lid margin thickening with diffuse or complete rounding [21]. Lid margin notching: 0 = no notching is observed, 1 = shallow dimpling of the lid margin and 2 = deep dimpling of the lid margin [21]. The posterior lid margin findings of vascularity Grade 0 = no or extremely minimal redness in lid margin conjunctiva and no telangiectasia crossing meibomian gland orifice. Grade 1 = redness in lid margin conjunctiva and no telangiectasia crossing meibomian gland orifices. Grade 2 = redness in lid margin conjunctiva and telangiectasia crossing meibomian gland orifices with a distribution of less than half of the full length of the lid and grade 3 = redness in lid margin conjunctiva and telangiectasia crossing meibomian gland orifices with a distribution of half or more of the entire length of the lid [21].

### Lipid profile

Venous blood samples were taken by phlebotomist for lipid profile analysis using the vacuum blood collection system (Becton Dickinson, Franklin Lakes, USA) gel separation tubes. Samples were collected after 12 hours of the overnight fast. The lipid profile was measured, including very low-density lipoprotein, low-density lipoprotein, high-density lipoprotein and triglycerides.

### Prolactin and testosterone assay

Serum prolactin and testosterone concentrations were determined using a commercially acquired kit (BioVendor–Laboratorní Medicína a.s. Czech Republic, Cat. No.: RCD023R) and (BioVendor–Laboratorn´ı Medic´ına a.s., Czech Republic, Cat. no.: RCD027R) for prolactin and testosterone, respectively. The determination of serum testosterone and prolactin concentrations followed the same protocol previously described in an earlier study [8]. Only 160 pregnant women had these hormones measured.

### Diagnosis of dry eye disease

Earlier studies have indicated that 96% of people with symptomatic dry eye exhibit one abnormal clinical sign [22,23]. Furthermore, the DEWS II report recommends symptomatology in addition to a minimum of one positive result of the markers of homeostasis comprising tear breakup time, ocular surface staining and tear osmolarity to constitute a diagnosis of dry eye disease [20].

Hence, an individual was diagnosed with the dry eye if the OSDI ≥ 13 and either the tear breakup time< 10 s or ocular surface staining (Oxford grading scale) ≥3 [20]. Afterward, if an individual had a Schirmer’s 1 test value ≤5, it was considered aqueous deficient dry eye and if MGD was diagnosed, the participant was considered as having an evaporative dry eye [20,23]. The mixed dry eye was diagnosed based on the presence of both MGD and abnormal Schirmer 1 test scores. An individual was classified as an unclassifiable dry eye if the Schirmer test was >5mm and no MGD diagnosis was made [23]. According to the MGD workshop (2011), for individuals who are “≥20 years old: a score of 1 for either meibum quality or expressibility is acceptable as normal; a score of 1 for both, or ≥1 for either, is abnormal with or without lid margin abnormalities” [18].

### Data analysis

Analyses were performed using SPSS version 21.0 (SPSS Inc., Chicago, IL) statistical package. The frequency of dry eye disease was determined among the cross-section of pregnant women, and the corresponding 95% confidence interval was estimated. The frequencies of evaporative dry eye, aqueous deficient dry eye, mixed dry eye and unclassifiable dry eye were estimated and their corresponding 95% confidence interval was estimated. A Chi-square test was used to determine whether the frequency of dry eye differs according to trimester. Binary logistic regression was used to determine the associated factors of dry eye in pregnant women. The one-way ANOVA with *post hoc* testing (Bonferroni) was conducted to determine differences in means across the clinical subtypes of dry eye. For 95% confidence level, P < .05 was considered statistically significant.

## Results and discussion

**Fig 1:**
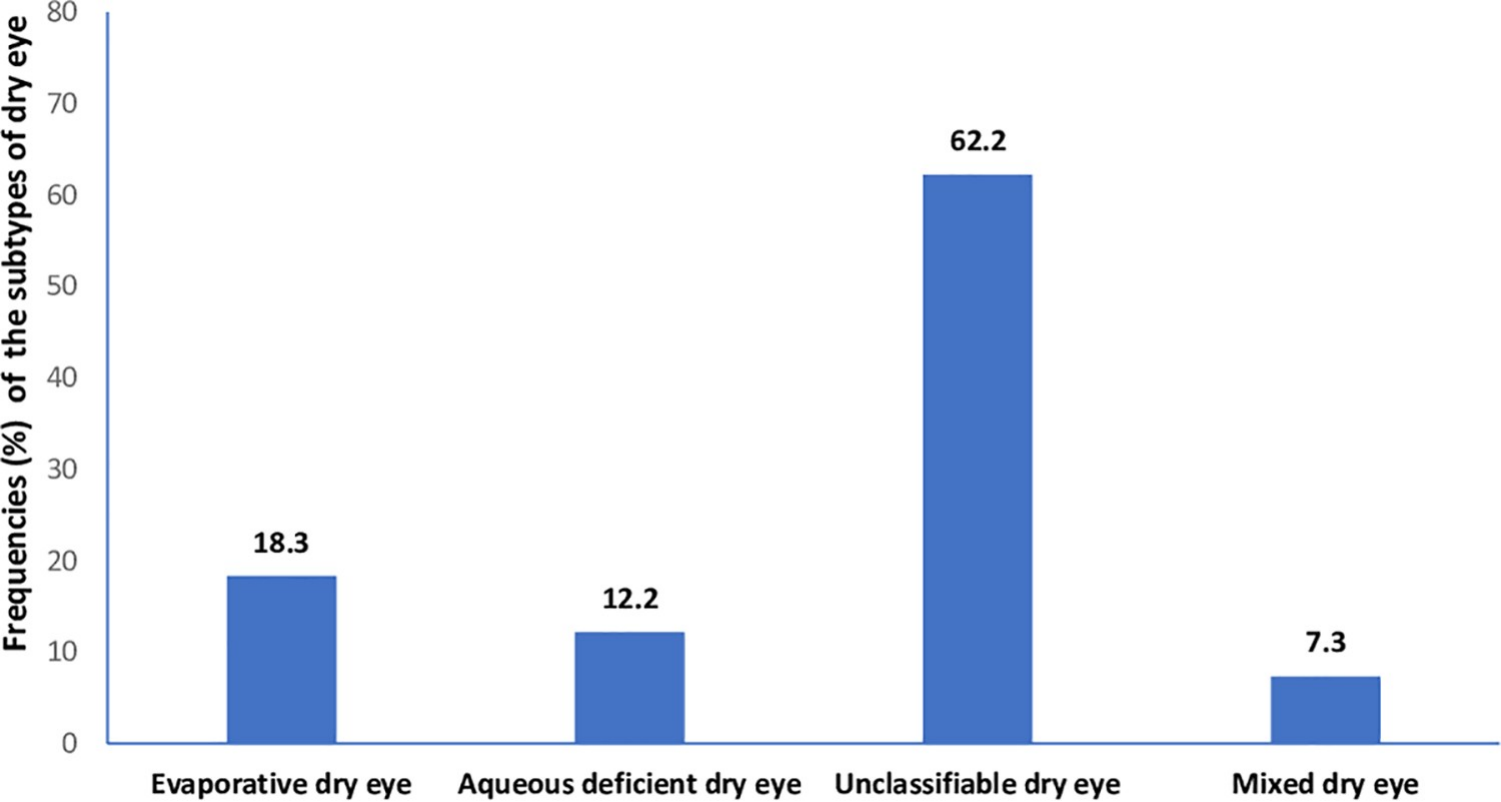
Frequencies of clinical subtypes of dry eye.

**Fig 2:**
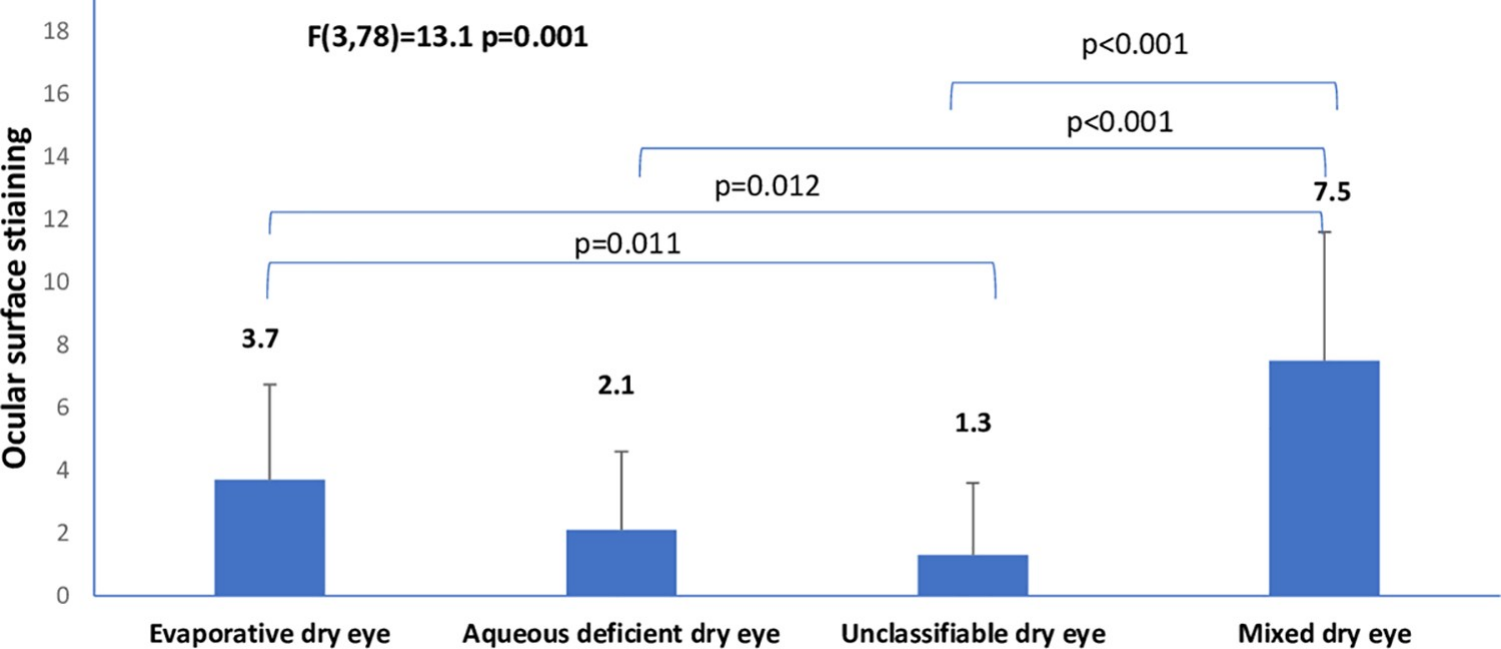
Means of ocular surface staining across clinical subtypes of dry eye.

**Fig 3:**
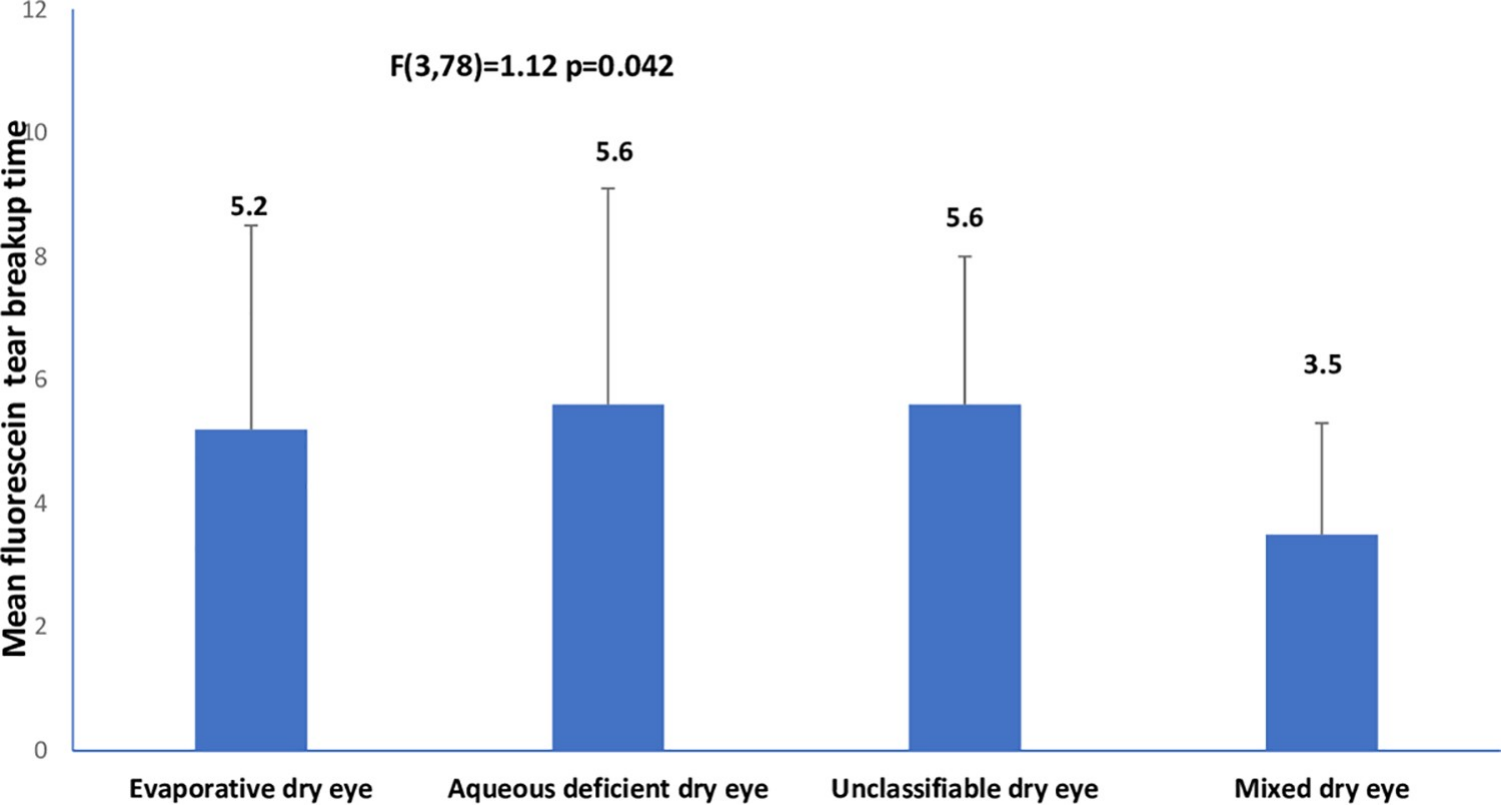
Means of fluorescein tear breakup time across clinical subtypes of dry eye.

**Fig 4:**
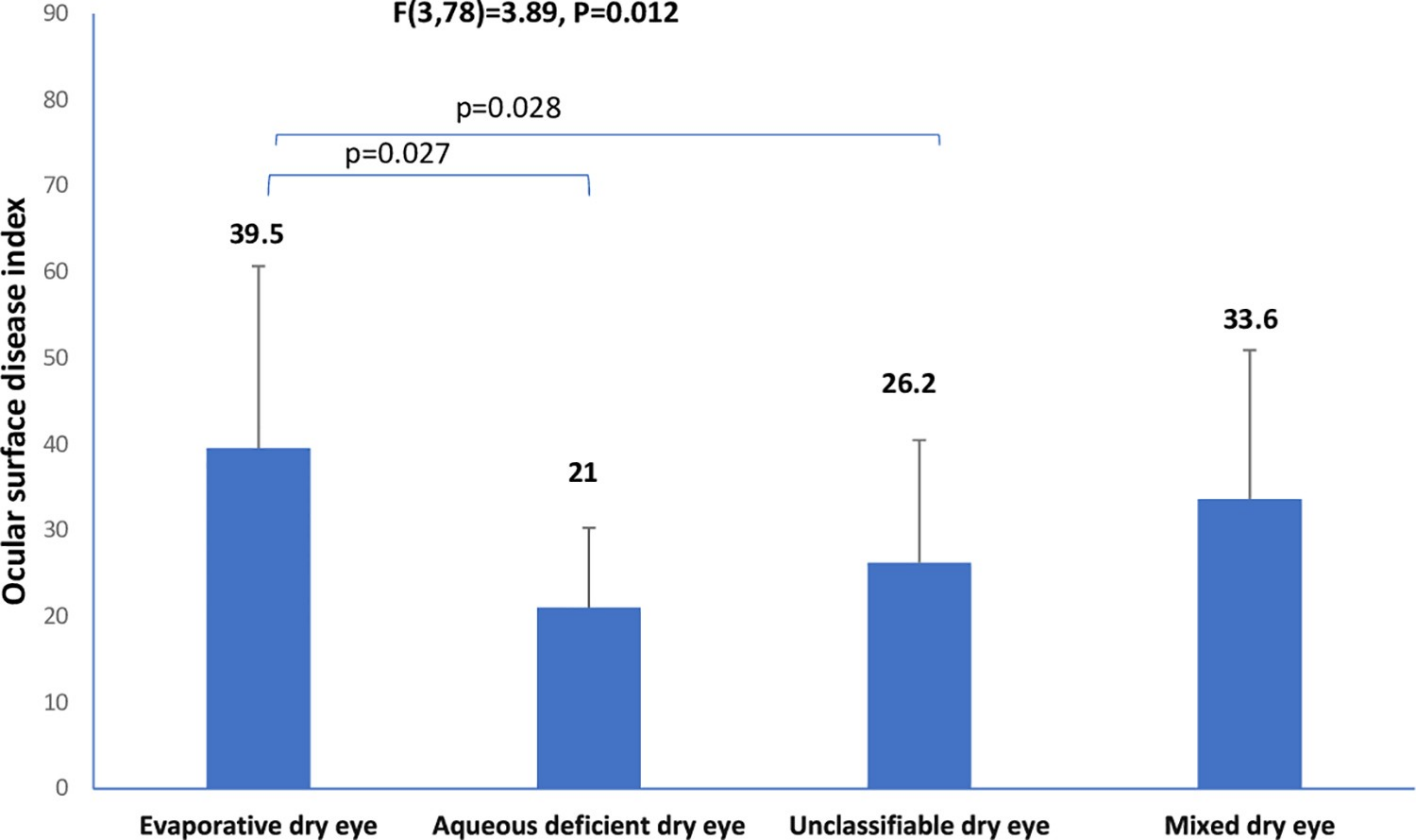
Means of ocular surface disease index across clinical subtypes of dry eye.

**Fig 5:**
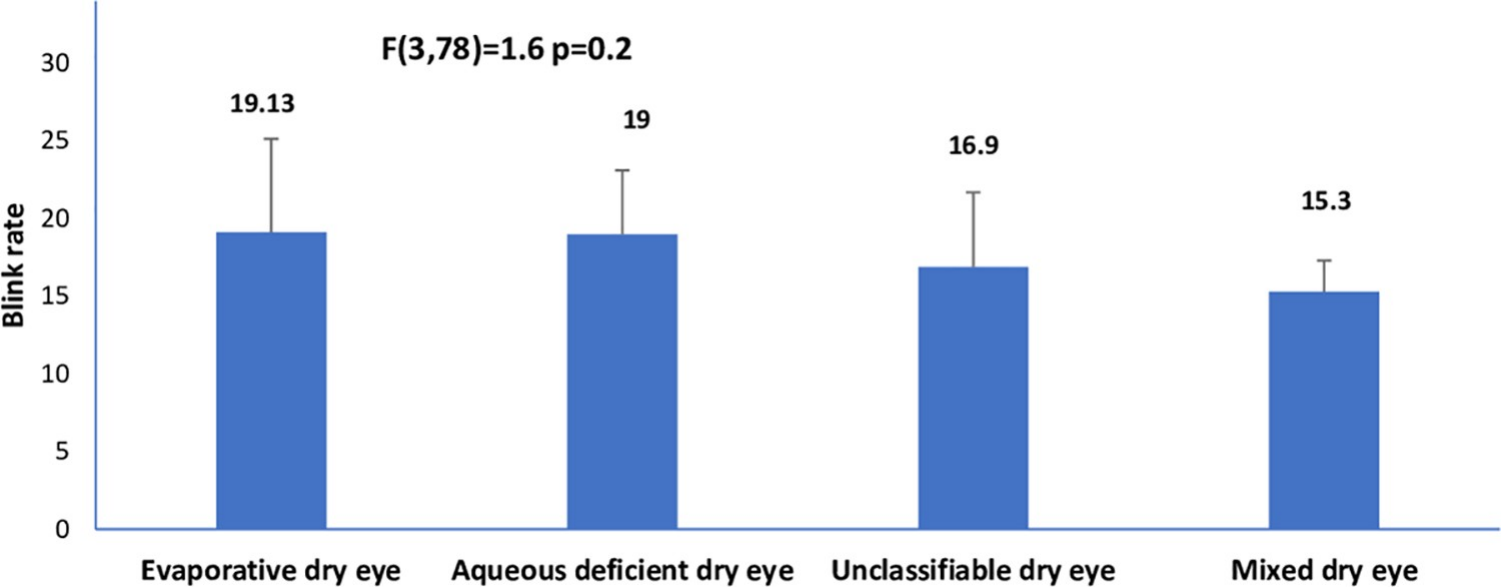
Means of blink rate across clinical subtypes of dry eye.

**Fig 6:**
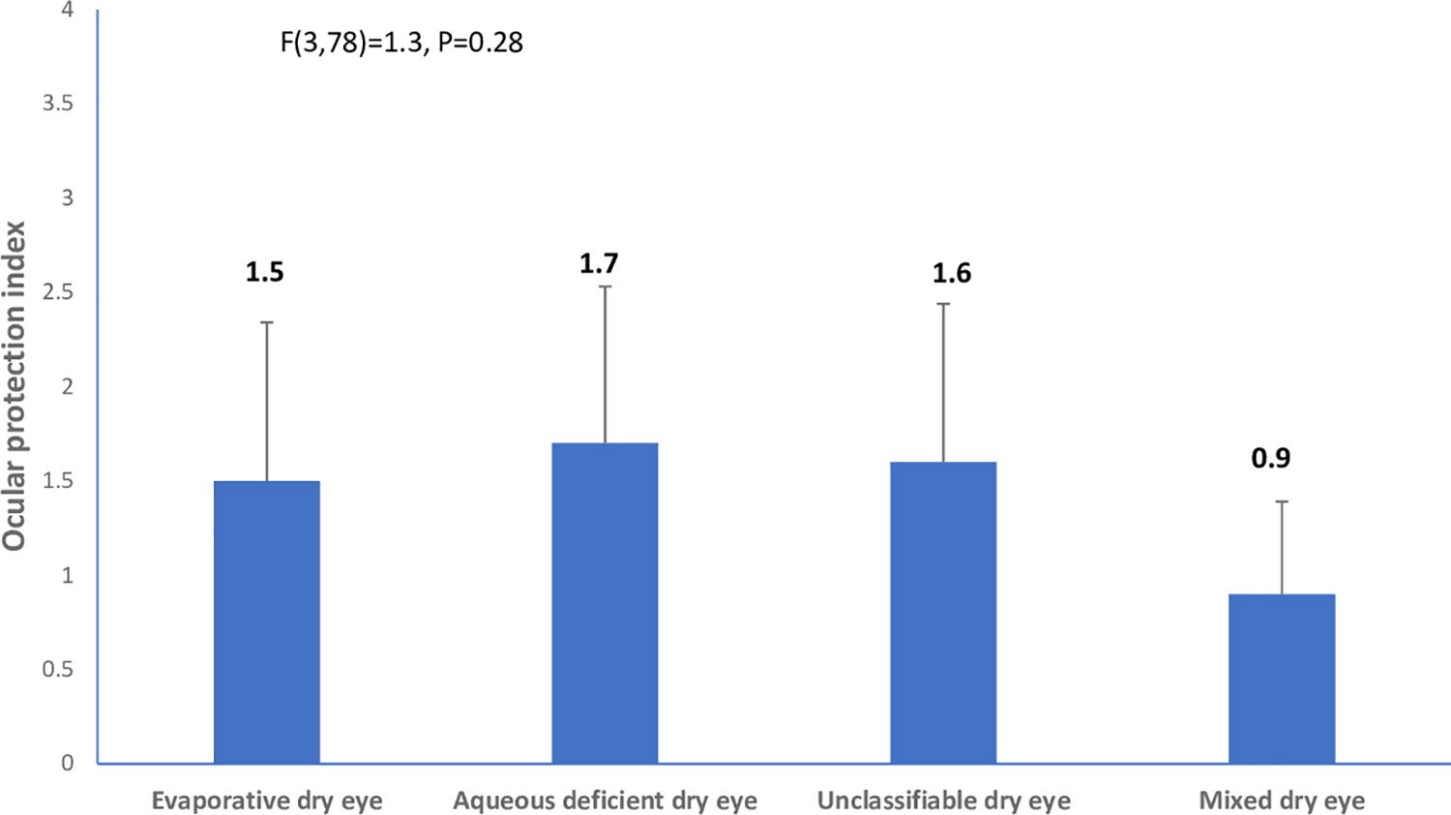
Means of ocular protection index across clinical subtypes of dry eye.

The mean age (SD) for the entire sample was 29. (±4.74) years (age range, 18–42 years). The prevalence of dry eye disease among the cohort of pregnant was 82/201 (40.8% 95% confidence interval 34.3%-47.3%). The descriptive characteristics of the cohort are shown in Table 1. Among the 82 pregnant women with dry eye disease, the frequencies of the clinical subtypes of dry eye were: evaporative dry eye [15/82(18.3%; 95% CI, 12.2%–25.2%)], aqueous deficient dry eye [10/82(12.2.%; 95% CI, 7.3%–18.3)], mixed dry eye [6/82(7.3%; 95% CI, 3.7%–11.0%)], and unclassified dry eye [51/82(62.2%; 95% CI, 52.4%–72.0%)]. The differences in tear breakup time, ocular surface staining, ocular surface disease index, ocular protection index and blink rate among the clinical subtypes of dry eye are illustrated in Figs 1–6.

**Table 1:** Descriptive characteristics of participants in the study.

Parameter	Minimum	Maximum	Means	Standard Deviation
Age	18.00	42.00	29.9550	4.73976
Body mass index	17.22	82.44	29.6366	8.35077
Gravidity	1.00	10.00	2.3532	1.37463
Gestational age	6.00	39.00	25.7750	9.07281
Diastolic blood pressure	50.00	90.00	66.3452	9.57477
Systolic blood pressure	90.00	150.00	108.0203	10.62533
Very low-density lipoprotein	7.00	61.00	26.4984	9.73084
Low-density lipoprotein	14.20	214.40	96.1312	42.73006
Total cholesterol concentration	55.00	343.00	189.7989	51.68853
Triglycerides	35.00	306.00	130.6032	48.97194
High density lipoproteins	16.00	150.00	67.9471	20.70608
Concentration of prolactin (ng/ml)	3.12	246.80	128.6356	45.80283
Concentration of testosterone (ng/ml)	35.04	100.65	69.0461	15.07128

There was no difference in the frequency of dry eye across the trimester (X^2^ = 4.79; df = 2 p = 0.091). Univariate binary logistic regression analysis showed that the following factors were not significantly associated with dry eye (P>0.05 for all): age, BMI, very low density lipoprotein, low-density lipoprotein, high-density lipoprotein and triglycerides, serum prolactin level, serum testosterone level, ocular protection index and blink rate. Only gestational age (P<0.05) was significantly associated with dry eye disease in pregnant women as shown in Table 2. However, a criterion of P < 0.09 was set to allow for multivariate logistic regression analysis. This criterion was based on the Wald’s test from logistic regression and a p-value cut-off point of 0.09 was chosen. This is because traditional levels such as 0.05 may fail in identifying variables known to be a confounder. In the iterative process of variable selection, covariates are removed from the model if they are not a confounder and non-significant. Besides these, only "gestational age" was significant with P< 0.05 in the univariate analysis so to obtain an adjusted ODDs ratio for "gestational age" we decided that any potentially significant factor should be included in the model. From Table 2 it can be seen that blink rate and high-density lipoprotein met this criterion and hence were included in the multivariate logistic regression. Multivariate binary logistic regression showed that only gestational age was statistically significant and predicted dry eye in the model. This is shown in Table 3.

**Table 2:** Univariate binary logistic regression analysis.

Parameter	Wald X^2^	P-value	ODDs ratio	95% confidence interval
Gravidity	0.042	0.84	1.022	0.83–1.26
BMI	0.019	0.89	1.02	0.8–1.3
Gestational age	5.53	0.019	1.039	1.006–1.072
Ocular protection index	1.54	0.22	1.221	0.89–1.7
Age	0.06	0.80	1.008	0.95–1.07
Very low-density lipoprotein (VLDL)	1.38	0.24	1.019	0.99–1.05
Low Density Lipoprotein(LDL)	0.538	0.46	1.003	1–1.009
High-density lipoprotein	3.11	0.08	0.98	0.97–1.001
Triglycerides	0.88	0.35	1.003	0.997–1.01
Total cholesterol	0.04	0.84	1.001	0.995–1.01
Testosterone	0.01	0.97	1.0	0.98–1.022
Prolactin	2.27	0.01	1.006	0.99–1.013
Blink rate	3.012	0.08	0.95	0.898–1.007

**Table 3:** Multivariate binary logistic regression analysis.

Parameter	Wald X^2^	P-value	ODDs ratio	95% confidence interval
High-density lipoprotein	2.29	0.11	0.989	0.97–1.003
Gestational age	4.78	0.029	1.037	1.004–1.072
Blink rate	2.44	0.12	095	0.889–1.012

Studies on dry eye disease have focused on the aging population, youthful population and postmenopausal women [24–26]. However, recent studies have indicated a high prevalence of dry eye in pregnant women [13] consistent with the finding of the current study. One study has reported reduced Schirmer test 1 scores in pregnant women compared to non-pregnant women [14]. However, studies on pregnant women suffer from relatively small sample sizes and these studies did not recruit pregnant women in the first trimester [13]. Furthermore, the definition for the dry eye was not entirely consistent with the dry eye definition endorsed by the dry eye workshop II [1]. This study utilized the definition of the dry eye workshop II to classify individuals as dry eye and reports a prevalence of 40.8% which is higher than the dry eye prevalence reported in many population-based and clinic-based studies. This high prevalence is based on a robust diagnostic criteria compared to that used in many typical dry eye studies [24,27]. This implies dry eye disease is very common in pregnancy regardless of gestational age and/or trimester [13,14]. Even animal studies show a decreased Schirmer’s test score and tear breakup time as well as increased Rose Bengal staining on the cornea in pregnant rats compared to non-pregnant rats [28]. For instance, Ding et al. conducted Schirmer’s test, tear breakup-time and ocular surface staining with Rose Bengal on pregnant and non-pregnant rabbits, and assessed the health of the ocular surface [29]. The tear breakup time and Schirmer test score were substantially reduced in the pregnant rabbits compared to the control group. Ibraheem et al. reported on healthy pregnant and non-pregnant women indicating that the Schirmer reading was significantly lower in the pregnant women compared to the control group [14].

Several reasons may account for the increase in dry eye disease in pregnancy. Researchers have demonstrated that dry eye disease during pregnancy may be due to damage to the lacrimal acinar cells through several mechanisms including but not limited to enhanced immune-reactivity of prolactin and adverse effects of transforming growth factor-beta 1 and epidermal growth factor on the ductal cells [13,30,31]. Evidence from animal studies shows changes in the expressions of aquaporin-4 and aquaporin-5 in pregnancy and these studies suggest that these alterations may instigate altered lacrimal gland secretion and eventually result in dry eye symptoms [29].

Potential causes of dry eye may be due to elevated estrogens and progesterone which mitigates androgens’ effects on the ocular surface through competitive antagonism [32,33]. There is also the potential for the downregulation of androgen receptors on the ocular surface of pregnant women further entrenching the desiccating effect of estrogen on the ocular surface [33]. Researchers have shown a moderate correlation between body fat and dry eye symptoms [34]. An association between adiposity measured by body fat percentage and symptoms of dry eye has been shown in the adult population [34]. Overall, pregnant women exhibit gains in weight, fat and body water content across pregnancy, and afterward, there is a decline in values during the postpartum period [35]. It may be plausible that an increase in adipose tissue in pregnancy may instigate or contribute to dry eye in pregnancy as reported in the general population [34]. Owing to the generally poor quality of life in pregnant women, treating dry eye disease in pregnant women will alleviate challenges with the quality of life. This is because dry eye symptoms impact the quality of life and the amelioration of dry eye symptoms is commensurate with an improved quality of life [3,36]. Factors associated with dry eye disease in pregnant women have been elusive as key dry eye risk factors such as age and sex are not important in pregnant women and other unrecognized factors may be important in the pregnancy-associated dry eye [24]. Many factors were explored in univariate and multivariate binary logistic regression analysis but only gestational age was significantly associated with dry eye in the multivariate analysis. From Table 2 it can be seen that even though the odds ratio for high-density lipoprotein and blink rate were not statistically significant in multivariate analysis but they appear to be protective for dry eye occurring in pregnant women. There is a trend of reduced odds of the dry eye occurring in pregnant women with increasing blink rate and high-density lipoprotein however this protection might be minor as they were not statistically significant. Even though trimester was not significantly associated with dry eye in this study nonetheless there was an increased odds of developing dry eye with increasing gestational age. This conundrum can be explained by the fact that two pregnancies could be in the same trimester but have an entirely different gestational age. Our results indicate that trimester may not be as important in the pregnancy-associated dry eye compared to gestational age. This finding is consistent with a longitudinal study that showed that dry eye increase from second to third trimester [13].

There was a high proportion of the participants with dry eye who could not be classified as aqueous deficient or evaporative dry eye. It implies that other etiological instigators in pregnancy might be responsible for dry eye clinical signs and symptoms. From Fig 2 it can be seen that participants with mixed dry eye had worse ocular surface staining or damage compared to aqueous deficient, evaporative or unclassifiable dry eye. This implies that increasing etiological instigators of dry eye are associated with more ocular surface damage. Furthermore, we found an increasing dry eye symptom in the evaporative dry eye compared to aqueous deficient dry eye or unclassifiable dry eye. In addition, the mixed dry eye has a high symptom score compared to aqueous deficient dry eye and unclassifiable dry eye. This implies that evaporative dry eye may be more symptomatic compared to aqueous deficient dry eye. This finding is consistent with the study by Lemp et al, which found subjects with evaporative dry eye demonstrated less ocular surface staining and higher OSDI scores compared with the mixed subtype and even aqueous deficient dry eye [37].

This study was a cross-sectional study hence the natural history of dry eye from the first to the third trimester was not explored. Besides, the potential risk factors were measured once hence we are unable to conclude whether they will remain not longitudinally associated with dry eye disease. Also, tear osmolarity measurement, a key homeostasis measure was not conducted in the study which could have provided a broader perspective to dry eye. However, tear breakup time and ocular surface staining are considered a pillar of tear film stability and ocular surface damage, hence may be adequate to diagnose dry eye in the absence of osmolarity testing [1,18,23,37,38].

In conclusion, the current study showed a high frequency of dry eye among a wide spectrum of pregnant women from the first to the third trimester and it is associated with increasing gestational age. The evaporative dry eye was more common compared to aqueous deficient dry eye, but most dry eye could not be classified.

## Supporting information

S1 File Table titles. (ZIP)

S2 File (SAV)

S3 File (SPV)
