# Correction: Dry eye, its clinical subtypes and associated factors in healthy pregnancy: A cross-sectional study

**DOI:** 10.1371/journal.pone.0263426

**Published:** 2022-01-27

**Authors:** Kofi Asiedu, Samuel Kyei, Madison Adanusa, Richard Kobina Dadzie Ephraim, Stephen Animful, Stephen Karim Ali-Baya, Belinda Akorsah, Mabel Antwiwaa Sekyere

In Table 2, the p-value for the Prolactin parameter should be 0.10. Please see the correct Table 2 here.

**Table 2:** Univariate binary logistic regression analysis.

Parameter	Wald X^2^	P-value	ODDs ratio	95% confidence interval
Gravidity	0.042	0.84	1.022	0.83–1.26
BMI	0.019	0.89	1.02	0.8–1.3
Gestational age	5.53	0.019	1.039	1.006–1.072
Ocular protection index	1.54	0.22	1.221	0.89–1.7
Age	0.06	0.80	1.008	0.95–1.07
Very low-density lipoprotein (VLDL)	1.38	0.24	1.019	0.99–1.05
Low Density Lipoprotein(LDL)	0.538	0.46	1.003	1–1.009
High-density lipoprotein	3.11	0.08	0.98	0.97–1.001
Triglycerides	0.88	0.35	1.003	0.997–1.01
Total cholesterol	0.04	0.84	1.001	0.995–1.01
Testosterone	0.01	0.97	1.0	0.98–1.022
Prolactin	2.27	0.10	1.006	0.99–1.013
Blink rate	3.012	0.08	0.95	0.898–1.007

There are errors in the axis labels in Fig 2 and Fig 3. Please see the correct Figs 2 and 3 here.

**Fig 2:**
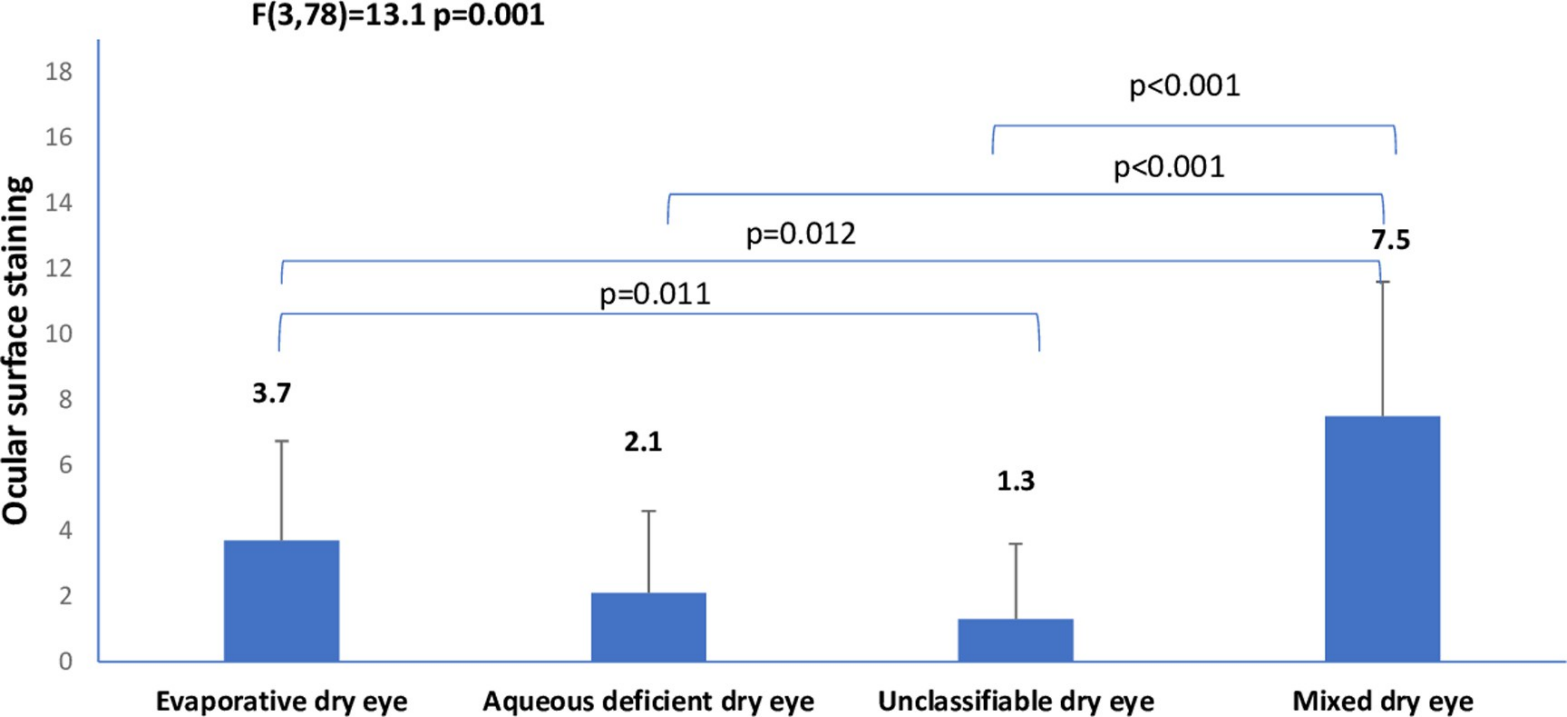
Means of ocular surface staining across clinical subtypes of dry eye.

**Fig 3:**
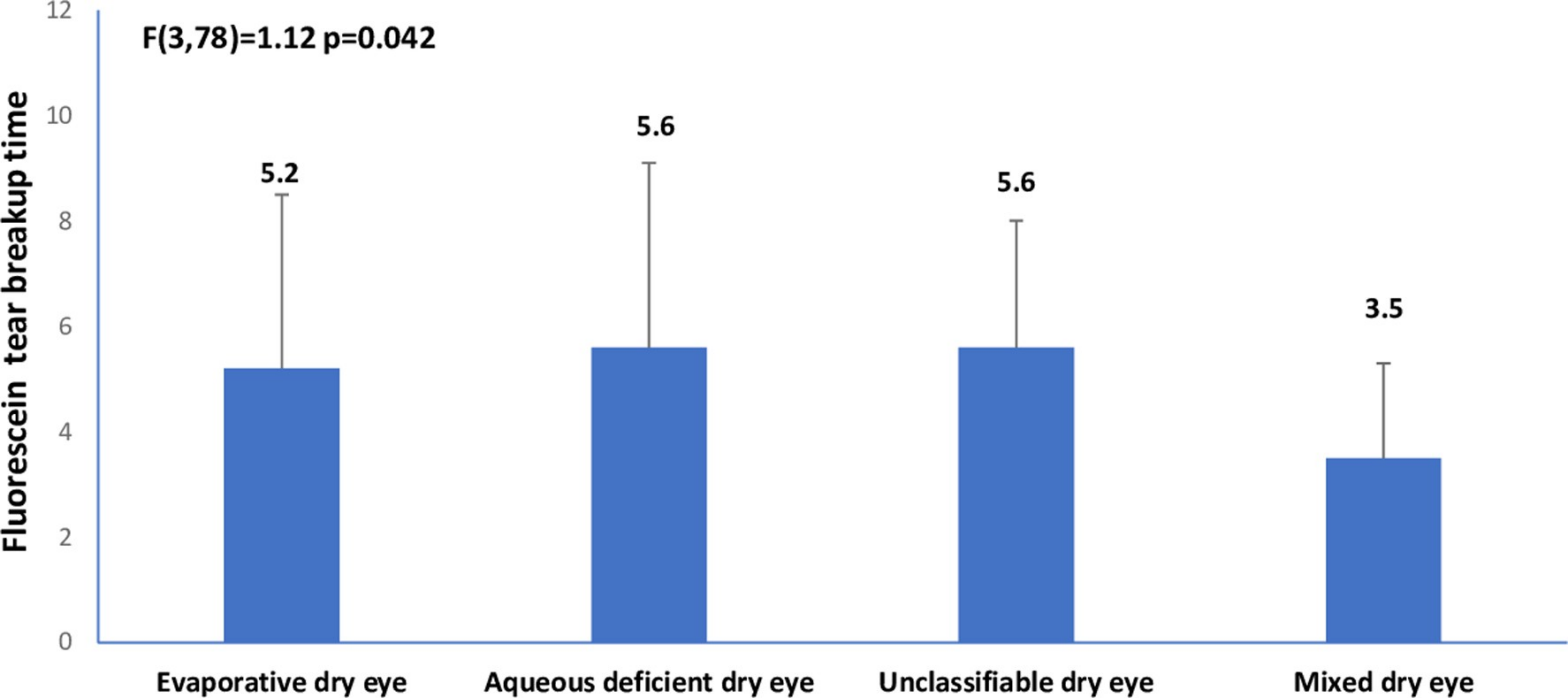
Means of fluorescein tear breakup time across clinical subtypes of dry eye.
